# Risk Factors for the Occurrence of Benign Paroxysmal Positional Vertigo: A Systematic Review and Meta-Analysis

**DOI:** 10.3389/fneur.2020.00506

**Published:** 2020-06-23

**Authors:** Jinbao Chen, Weisong Zhao, Xuejing Yue, Ping Zhang

**Affiliations:** ^1^Department of Pediatrics, The First Clinic College of Xinxiang Medical University, Xinxiang, China; ^2^School of Basic Medicine, Xinxiang Medical University, Xinxiang, China; ^3^Department of Neurology, The First Affiliated Hospital of Xinxiang Medical University, Xinxiang, China

**Keywords:** benign paroxysmal positional vertigo, risk factors, occurrence, systematic review, meta-analysis

## Abstract

**Background and Purpose:** The lifetime prevalence of benign paroxysmal positional vertigo (BPPV) is high, especially in the elderly. Patients with BPPV are more susceptible to ischemic stroke, dementia, and fractures, severely reducing quality of life of patients. Many studies have analyzed risk factors for the occurrence of BPPV. However, the results of these studies are not identical. We performed this meta-analysis to determine potential risk factors associated with the occurrence of BPPV.

**Methods:** PubMed, EMBASE, and the Cochrane Library (January 2000 through March 2020) were systematically searched for eligible studies analyzing risk factors for the occurrence of BPPV. Reference lists of eligible studies were also reviewed. We selected observational studies in English with a control group and sufficient data. Pooled odds ratios (ORs) or the mean differences (MDs) and 95% confidence intervals (CIs) were calculated to measure the impacts of all potential risk factors. Heterogeneity among studies was evaluated using the *Q*-test and *I*^2^ statistics. We used the random-effect model or the fixed-effect model according to the heterogeneity among the included studies.

**Results:** We eventually included 19 studies published between 2006 and 2019, including 2,618 patients with BPPV and 11,668 participants without BPPV in total. In this meta-analysis, the occurrence of BPPV was significantly associated with female gender (OR = 1.18; 95% CI, 1.05–1.32; *P* = 0.004), serum vitamin D level (MD = −2.12; 95% CI, −3.85 to −0.38; *P* = 0.02), osteoporosis (OR = 2.49; 95% CI, 1.39–4.46; *P* = 0.002), migraine (OR = 4.40; 95% CI, 2.67–7.25; *P* < 0.00001), head trauma (OR = 3.42; 95% CI, 1.21–9.70; *P* = 0.02), and total cholesterol level (MD = 0.32; 95% CI, 0.02–0.62; *P* = 0.03).

**Conclusion:** Female gender, vitamin D deficiency, osteoporosis, migraine, head trauma, and high TC level were risk factors for the occurrence of BPPV. However, the effects of other risk factors on BPPV occurrence need further investigations.

## Introduction

Benign paroxysmal positional vertigo (BPPV) is one of the most common types of vestibular vertigo, accounting for ~17–42% of patients with vertigo (1, 2). Patients suffering from BPPV are characterized by transient episodes of vertigo provoked by head position changes (3). The lifetime prevalence of BPPV is estimated at 2.4%, and the 1-year prevalence of BPPV in the elderly is much higher than that in other age groups (4). In addition, some studies have suggested that patients with BPPV were more susceptible to future ischemic strokes, dementia, and fractures, which severely reduces quality of life of patients, especially in the elderly (5–7). Thus, identifying potential risk factors for the occurrence of BPPV can help prevent this disease. Furthermore, some serum indicators may also help improve the clinical misdiagnosis of some atypical BPPV.

Although canalith repositioning maneuver is an effective treatment for BPPV, nearly 50% of patients experienced at least one recurrence in 2 years after treatment (8). Many of the risk factors investigated in this meta-analysis, such as hypertension and migraine, may also be risk factors for BPPV recurrence, which may help improve the treatment and prognosis of this disease (9).

However, the underlying causes of BPPV remain unclear. In recent decades, many studies have investigated risk factors for the occurrence of BPPV, including female gender, serum vitamin D deficiency, osteoporosis, vascular risk factors, head trauma, and other potential risk factors (10–26). However, there are some controversies among these studies. The primary purposes of this meta-analysis are to identify the underlying risk factors for BPPV occurrence and summarize the evidence for screening high-risk populations to reduce the incidence of BPPV.

## Methods

### Literature Search Strategy

The electronic databases PubMed, EMBASE, and the Cochrane Library (January 2000 through March 2020) were systematically searched by two researchers (JB Chen and WS Zhao) for eligible observational studies analyzing risk factors for the occurrence of BPPV. The MeSH terms “Risk Factors,” “Benign Paroxysmal positional vertigo,” and all related free words were combined to search relevant literature as comprehensively as possible. Reference lists of all eligible studies were also reviewed to identify other potentially relevant studies.

### Selection Criteria

Articles included in this meta-analysis must meet the following criteria: (1) clearly define the experimental group (patients diagnosed with BPPV) and the control group (participants or patients without any history of vertigo); (2) all BPPV patients included in studies were diagnosed by a characteristic history of recurrent positional vertigo or a typical nystagmus during Dix-Hallpike tests or Roll test; (3) reported sufficient data on risk factors investigated in our meta-analysis; (4) the outcome was BPPV; (5) case–control studies, cohort studies, or other observational English studies analyzing relevant risk factors for occurrence of BPPV. The following studies were excluded from this meta-analysis: (1) sufficient information could not be obtained; (2) the outcome was the recurrence of BPPV, not the occurrence of BPPV.

### Data Extraction and Quality Assessment

Two reviewers (JB Chen and WS Zhao) independently assessed the quality of each study included in this meta-analysis using the Newcastle Ottawa Scale (27). Studies were evaluated according to three dimensions including selection, comparability, and outcome (cohort studies) or exposure (case–control studies). Any discrepancies between the two reviewers were resolved through discussion with another author (XJ Yue). The total NOS scores of all included articles are shown in Table 1. Studies with NOS scores ≥7 were considered high quality.

**Table 1:** Baseline characteristics of each study included in this meta-analysis.

**Reference**	**Study region**	**Study design**	**Sample size (case/control)**	**Mean age (SD/IQR)**	**BMI (mean ± SD)**	**Risk factors included**	**NOS score**
Karataş et al. (12)	Turkey	Case–control study	78/78	51.4 ± 12.2/48.9 ± 12.5	26.2 ± 3.0/26.0 ± 2.3	F1, F2, F3, F4, F9, F10, F15	7
Yuan et al. (20)	Beijing, China	Case–control study	240/72	62.4 ± 12.5/63.5 ± 11.9	24.9 ± 2.9/25.6 ± 2.8	F1, F2, F12	7
Celikbilek et al. (26)	Turkey	Case–control study	50/40	33.4 ± 6.15/32 ± 6.74	25.31 ± 2.35/24.47 ± 2.77	F1, F2, F12	6
Yang et al. (14)	Korean	Case–control study	130/130	54.9 ± 12.2/54.9 ± 12.2	NA	F1, F2, F3, F4, F5	7
Işik et al. (10)	Turkey	Case–control study	64/63	NA	NA	F1, F3	6
Cai et al. (17)	Lanzhou, China	Case–control study	154/100	Median 37/37 (IQR 31–43/30–43)	Median 25.3/24.5 (IQR 24.1–27.0/24.3–27.5)	F1, F13, F14, F15	5
Jeong et al. (15)	Korean	Case–control study	100/192	61.8 ± 11.6/60.3 ± 11.3	24.9 ± 3.4/23.3 ± 3.6	F1, F2, F3, F4, F5, F9, F10, F15	9
Ding et al. (25)	Lanzhou, China	Cross-sectional study	174/348	Median 61/61 (IQR 54–69/54–69)	Median 25.8/26.0 (IQR 24.3–27.4/24.4–27.6)	F1, F9, F10, F11, F13, F14, F15	7
von Brevern et al. (4)	Germany	Cross-sectional study	53/6136	NA	NA	F1, F6, F7, F9, F10, F11, F13	5
Jeong et al. (23)	Korean	Case–control study	209/202	59.8 ± 12.5/56.3 ± 8.6	NA	F1, F2, F4, F5, F9, F10, F11, F13, F14	8
Han et al. (22)	Ningbo, China	Case–control study	85/80	63.5 ± 9.72/63.9 ± 9.87	23.8 ± 3.02/23.6 ± 3.29	F2, F3, F4, F5, F9, F10	6
Wu et al. (24)	Ningbo, China	Case–control study	78/126	58.4 ± 11.4/58.5 ± 10.3	22.69 ± 3.34/23.48 ± 3.28	F2, F4, F5, F9, F10, F15	6
Wu et al. (11)	Ningbo, China	Case–control study	60/92	59.4 ± 13.2/62.1 ± 10.6	23.6 ± 2.8/23.9 ± 2.8	F2, F3, F4, F5, F9, F10, F13, F14	7
Zhang et al. (19)	Zhengzhou, China	Case–control study	104/88	73/71 (Range 65–88/65–84)	NA	F1, F9, F10	5
Yang et al. (18)	Shanghai, China	Case–control study	50/52	NA	22.62 ± 2.47/24.74 ± 12.7	F3, F9, F10	7
Chang et al. (13)	Taiwan, China	Case–control study	768/1,536	57 ± 15/57 ± 15	NA	F1, F2, F4, F6, F7, F8, F9, F10, F11	9
Sunami et al. (16)	Japan	Case–control study	156/155	56.27 ± 14.63/56.39 ± 15.66	NA	F1, F2, F13, F14	6
Pan et al. (28)	Beijing, China	Case–control study	120/60	61.30 ± 9.20/61.32 ± 9.54	NA	F1, F2, F9, F10, F12, F13, F14	8
Kim et al. (29)	Korean	Case–control study	23/2,196	54.09 ± 19.13/52.60 ± 18.43	NA	F1, F2, F8	7

*NA, not available; SD, standard deviation; IQR, interquartile range; Risk Factors: F1, female gender; F2, age; F3, serum vitamin D level; F4, osteoporosis; F5, osteopenia; F6, migraine; F7, stroke; F8, head trauma; F9, hypertension; F10, diabetes mellitus; F11, hyperlipidemia; F12, TC level; F13, smoking; F14, drinking; F15, regular exercise*.

A standardized pre-extraction form was used to extract available data, including study characteristics, sample demographic information, medical comorbidities, and serum indicators. For each risk factor, we performed a detailed analysis and compared their definitions in the original literature. Data extraction was independently completed by the same two reviewers according to the revised extraction form from January 2020 to February 2020. All disagreements between the two reviewers were fully discussed, and furthermore a third reviewer (XJ Yue) was consulted for unresolved discrepancies to reach a consensus. The following data were extracted for each included study: (1) Study characteristics: fist author, study region, sample size, publication year, and study design (case–control or cross-sectional study); (2) sample demographic information: gender, age (mean ± SD), body mass index (BMI), smoking, drinking, and regular exercise; (3) medical comorbidities of participants: osteoporosis, osteopenia, migraine, stroke, head trauma, hypertension (HTN), diabetes mellitus (DM), and hyperlipidemia; (4) serum indicators: total cholesterol level (TC) (mmol/L) and serum vitamin D level (ng/ml).

### Statistical Analysis

The impacts of all potential risk factors on the occurrence of BPPV were measured by calculating odds ratios (ORs) or mean differences (MDs) and 95% confidence intervals (CIs). ORs were calculated for categorical variables including female gender, osteoporosis, osteopenia, migraine, stroke, head trauma, hypertension, DM, hyperlipidemia, smoking, drinking, and regular exercise. MDs were calculated for continuous variables including age, serum vitamin D level, and TC level. Heterogeneity among studies was tested and quantified using the Cochrane *Q*-test and *I*^2^ statistics. A fixed-effect model was used when heterogeneity was not significant (*I*^2^ < 50%) and a random-effect model was used when heterogeneity was significant (*I*^2^ > 50%) (30). In addition, funnel plots of some risk factors were used to assess the publication bias in included studies. All statistical analyses were performed using the Review Manager 5.3 software.

## Results

### Study Selection and Characteristics

The literature search produced a total of 256 records. Six additional records were identified through screening the reference lists of each study included in this meta-analysis. After 49 duplicates were removed, we further excluded 158 records through screening the titles/abstracts. The remaining 55 studies were assessed by reviewing the full text in detail. Finally, 19 studies published between 2006 and 2019 were included in our meta-analysis. A flow diagram of the literature selection was present in Supplemental Figure 1. A total of 14,286 participants were included in this meta-analysis, including 2,618 patients with BPPV and 11,668 controls without BPPV. Most studies were conducted in Asia. Furthermore, 5 studies were prospective (17–19, 23, 26), 12 were retrospective (10–16, 20, 22, 24, 28, 29), and 2 were cross-sectional (4, 25). In addition, the NOS scores of each study ranged from 5 to 9, indicating a medium and high quality of all included studies. Baseline characteristics of each study and pooled results for each risk factor were summarized in Tables 1, 2, respectively. Funnel plots of some risk factors showed that no significant publication bias was found in the included studies (Supplemental Figures 2–5). A total of 15 potential risk factors were assessed including female gender, age, osteoporosis, osteopenia, serum vitamin D level, migraine, stroke, head trauma, HTN, DM, hyperlipidemia, TC level, smoking, drinking, and regular exercise.

**Table 2:** The pooled results for each risk factor included in this meta-analysis.

**Risk factors**	**Number of studies**	**Number of participants**	**Pooled results**			**Heterogeneity** ***I***^****2****^
			**OR/MD**	**95% CI**	***P* value**		***P* value for heterogeneity**	**Analytical effect model**
Female gender	15	13,819	1.18	1.05, 1.32	0.004	49%	0.02	Fixed-effect model
Age	13	7,056	0.56^*^	−0.17,1.29	0.13	20%	0.24	Fixed-effect model
Serum vitamin D level	7	1,254	−2.12^*^	−3.85, −0.38	0.02	75%	0.0006	Random-effect model
Osteoporosis	8	3,944	2.49	1.39, 4.46	0.002	79%	<0.0001	Random-effect model
Osteopenia	6	1,484	1.11	0.76, 1.62	0.59	63%	0.02	Random-effect model
Migraine	2	8,493	4.40	2.67, 7.25	<0.00001	0%	0.81	Fixed-effect model
Stroke	2	8,493	3.58	0.43, 29.93	0.24	93%	0.0002	Random-effect model
Head trauma	2	4,523	3.42	1.21, 9.70	0.02	67%	0.08	Random-effect model
Hypertension	12	10,869	1.26	0.97, 1.62	0.08	65%	0.001	Random-effect model
Diabetes mellitus	12	10,869	1.04	0.86, 1.25	0.71	18%	0.27	Fixed-effect model
Hyperlipidemia	4	9,426	1.50	0.88, 2.53	0.13	86%	0.0001	Random-effect model
TC level	3	582	0.32^*^	0.02, 0.62	0.03	66%	0.05	Random-effect model
Smoking	7	8,019	0.59	0.33, 1.04	0.07	80%	<0.0001	Random-effect model
Drinking	6	1,830	0.64	0.29, 1.43	0.28	89%	<0.00001	Random-effect model
Regular exercise	5	1,428	1.08	0.79, 1.47	0.63	0%	0.84	Fixed-effect model

*OR, odds ratio; MD, mean difference; CI, confidence intervals; TC, total cholesterol*;

* *, MD*.

### Female Gender

**Figure 1:**
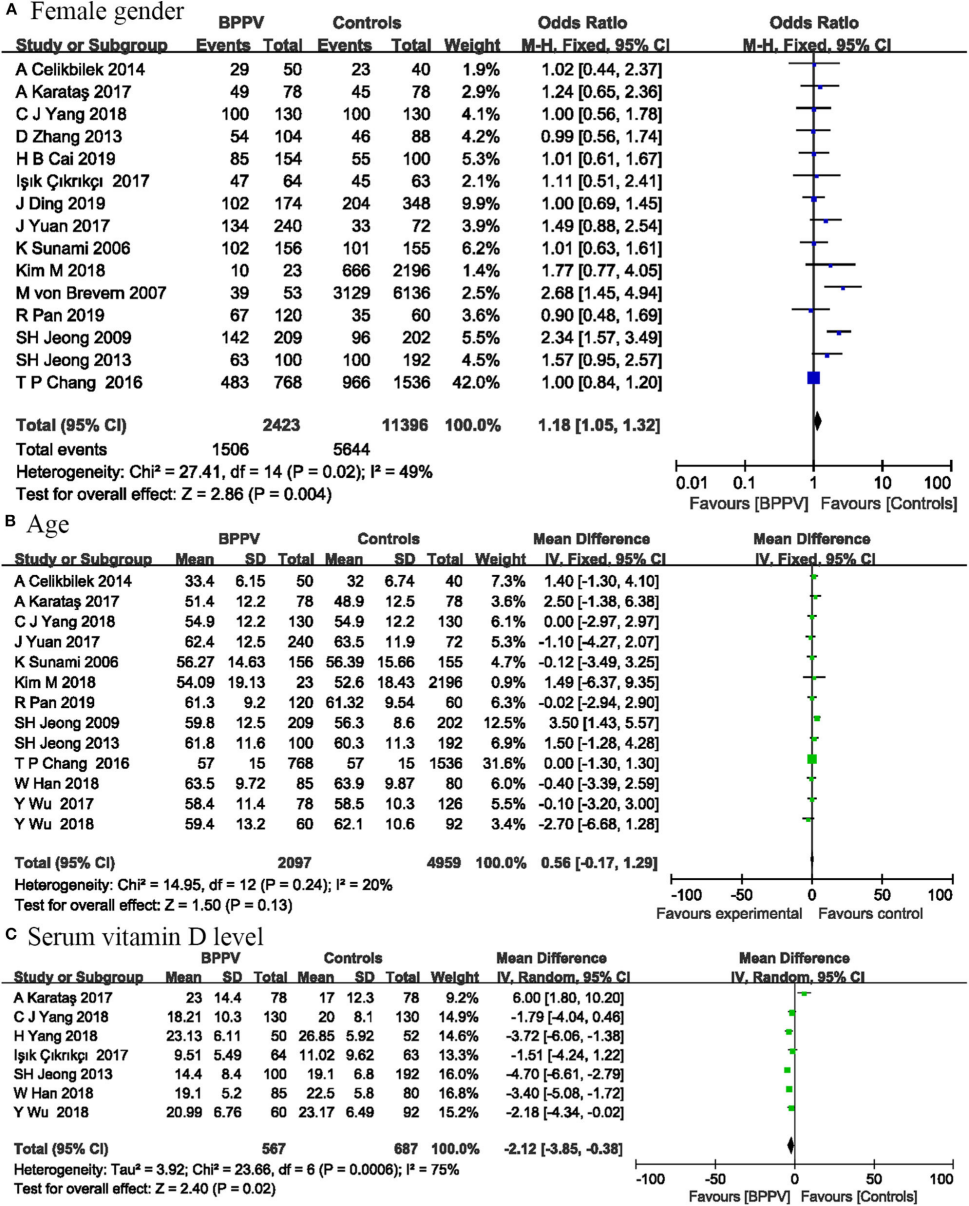
Forest plot of female gender **(A)**, age **(B)**, and serum vitamin D level **(C)**.

Fifteen studies involving 13,819 participants analyzed the relationship between female gender and the occurrence of BPPV. Four studies were not included in this risk factor analysis, because the participants in these studies were all male or female. The pooled results showed that female had a slightly higher risk of BPPV compared with male (OR = 1.18; 95% CI, 1.05–1.32; *P* = 0.004) (Figure 1). We used a fixed-effect model, because the statistical heterogeneity between these studies was not significant (*I*^2^ = 49%; *P* = 0.02).

### Age

Thirteen studies including 7,056 participants reported sufficient data between age and the occurrence of BPPV. The pooled results showed that age was not associated with BPPV occurrence (MD = 0.56; 95% CI, −0.17–1.29; *P* = 0.13) (Figure 1). These results may be partly due to the fact that many included studies controlled the age between the experimental and control groups. We used a fixed-effect model, because the statistical heterogeneity between these studies was not significant (*I*^2^ = 20%; *P* = 0.24).

### Serum Vitamin D Level

Seven studies including 1,254 participants measured serum vitamin D level to investigate the relationship between serum vitamin D level and BPPV occurrence. Significant relationship was found between serum vitamin D level and BPPV in our analysis. The vitamin D level was lower in patients with BPPV than in controls (MD = −2.12; 95% CI, −3.85 to −0.38; *P* = 0.02) (Figure 1). Statistical heterogeneity was significant (*I*^2^ = 75%; *P* = 0.0006). As shown in Supplemental Figure 6, the results of sensitivity analysis were consistent with previous analysis (MD = −3.09; 95% CI, −3.95 to −2.23; *P* < 0.00001; *I*^2^ = 22%; *P* = 0.27).

### Bone Mineral Density

**Figure 2:**
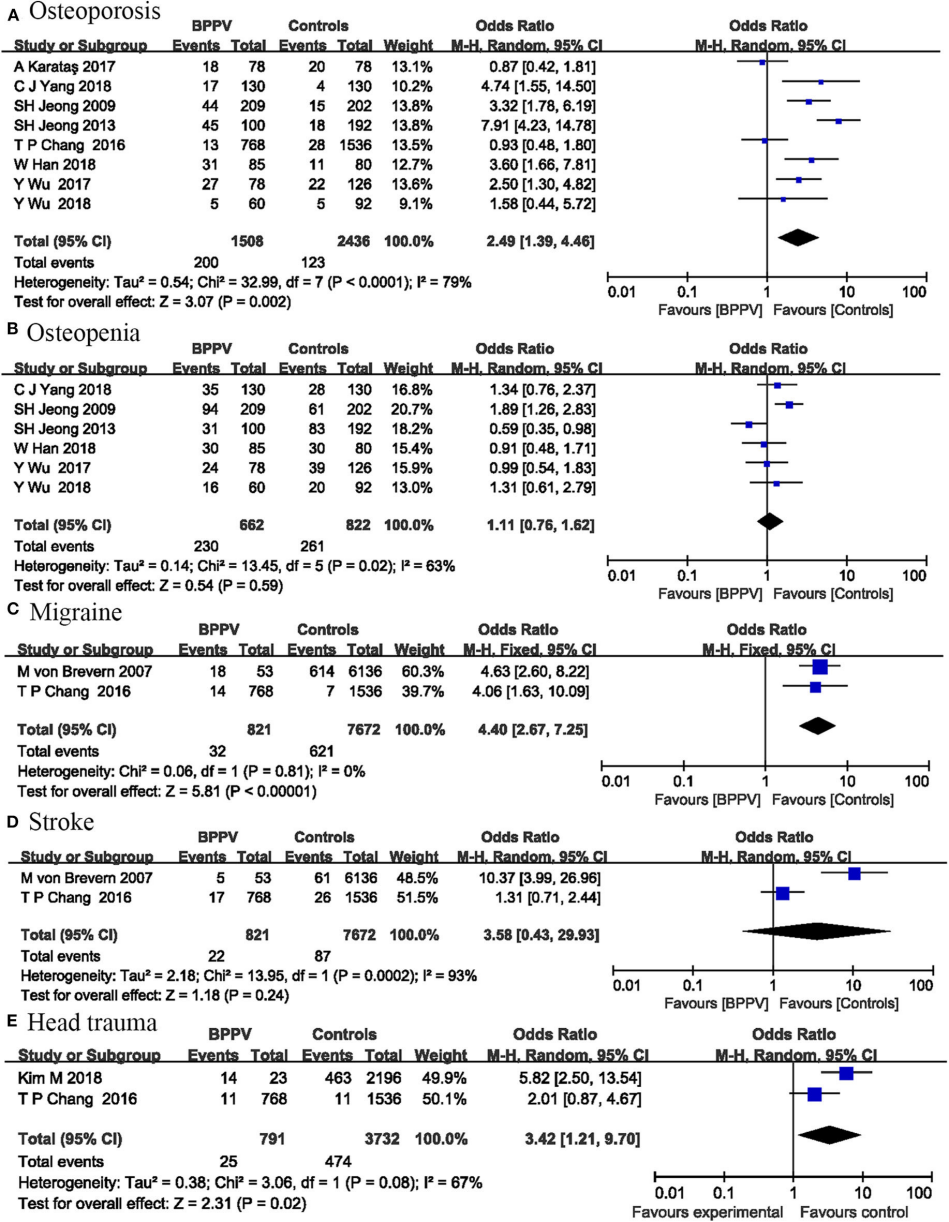
Forest plot of osteoporosis **(A)**, osteopenia **(B)**, migraine **(C)**, stroke **(D)**, and head trauma **(E)**.

Bone mineral density measurements were expressed as *T* scores and we specifically analyzed the effects of osteoporosis and osteopenia on BPPV. Osteopenia was defined as −2.5 < *T* score < -1.0, and osteoporosis was defined as *T* score ≤ -2.5. Eight studies including 3,944 participants investigated the effects of osteoporosis on the occurrence of BPPV. Our analysis indicated that osteoporosis was a risk factor for BPPV occurrence (OR = 2.49; 95% CI, 1.39–4.46; *P* = 0.002) (Figure 2). The *I*^2^-value was 79%, suggesting significant heterogeneity among these studies. Six studies involving 1,484 participants were included in osteopenia analysis. No significant relationship was found between osteopenia and BPPV (OR = 1.11; 95% CI, 0.76–1.62; *P* = 0.59) (Figure 2). The *I*^2^-value was 63%.

### Migraine and Stroke

Two studies including 8,493 participants investigated the relationship between migraine and BPPV occurrence. Our analysis indicated that migraine was a risk factor for BPPV occurrence (OR = 4.40; 95% CI, 2.67–7.25; *P* < 0.00001**)** (Figure 2). No heterogeneity was detected between these studies (*I*^2^ = 0%; *P* = 0.81).

The same two studies also analyzed the correlation between stroke and the occurrence of BPPV. The pooled results showed no significant correlation between BPPV and stroke (OR = 3.58; 95% CI, 0.43–29.93; *P* = 0.24) (Figure 2), with significant heterogeneity between the two studies (*I*^2^ = 93%; *P* = 0.0002).

### Head Trauma

Two studies including 4,523 participants investigated the relationship between head trauma and BPPV occurrence. Our analysis indicated that head trauma was a risk factor for BPPV occurrence (OR = 3.42; 95% CI, 1.21–9.70; *P* = 0.02) (Figure 2). The *I*^2^-value was 67%, indicating significant heterogeneity between the two studies.

### Hypertension

**Figure 3:**
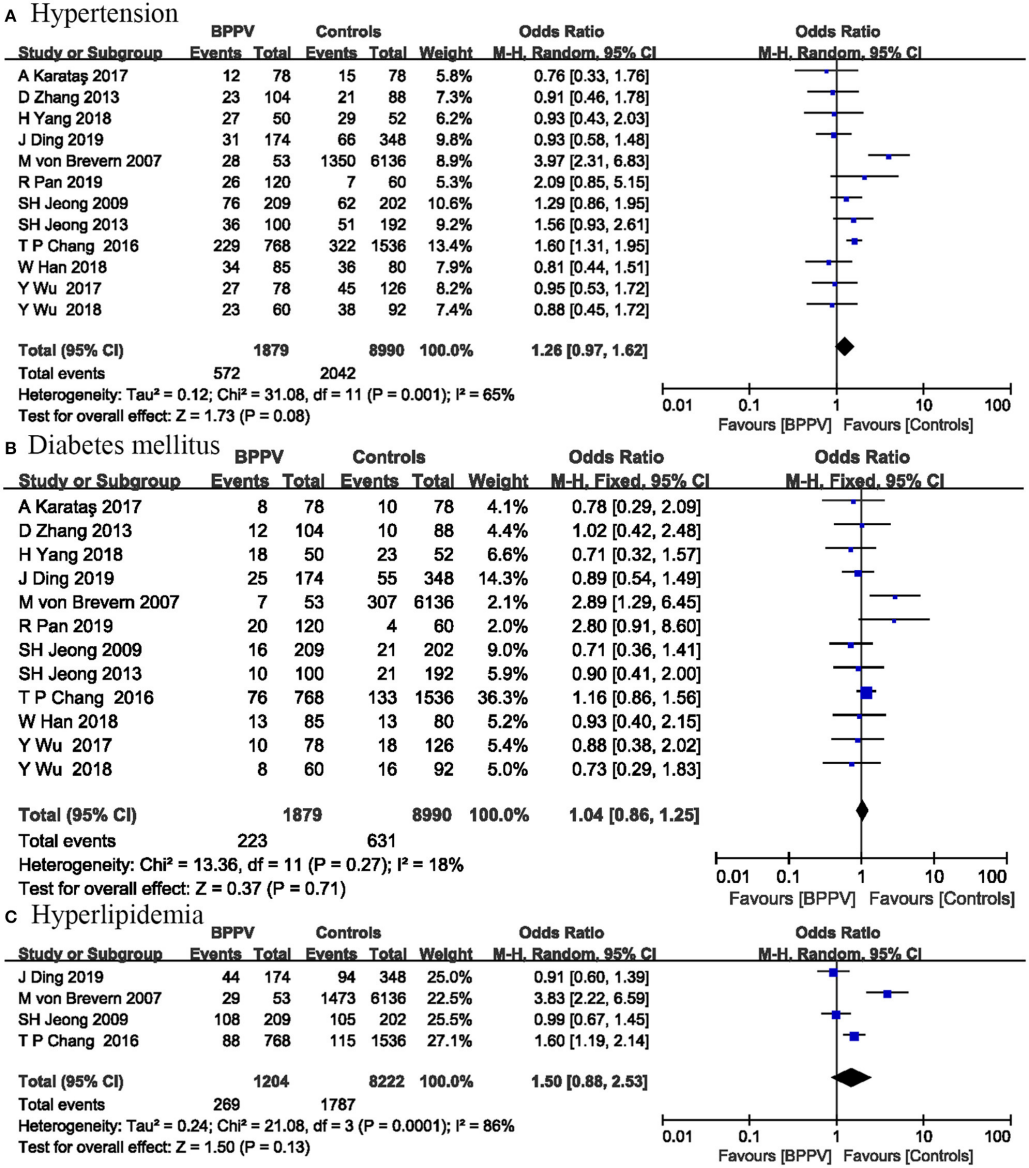
Forest plot of hypertension **(A)**, diabetes mellitus **(B)**, and hyperlipidemia **(C)**.

Twelve studies including 10,869 participants evaluated the effects of hypertension on the onset of BPPV. The pooled results suggested no significant association between BPPV and hypertension (OR = 1.26; 95% CI, 0.97–1.62; *P* = 0.08) (Figure 3). This risk factor was analyzed by a random-effect model (*I*^2^ = 65%; *P* = 0.001). Significant heterogeneity between studies limited the accuracy of the results.

### Diabetes Mellitus

Twelve studies including 10,869 participants reported the relationship between DM and BPPV occurrence. The pooled evidence showed that DM was not associated with BPPV occurrence (OR = 1.04; 95% CI, 0.86–1.25; P = 0.71) (Figure 3). No significant heterogeneity was detected among these studies, and a fixed-effect model was used (*I*^2^ = 18%; *P* = 0.27).

### Hyperlipidemia and TC Level

Four studies including 9,426 participants investigated the influence of hyperlipidemia on the occurrence of BPPV. Our analysis showed no significant association between hyperlipidemia and BPPV occurrence (OR = 1.50; 95% CI, 0.88–2.53; *P* = 0.13) (Figure 3). The *I*^2^-value was 86%, so a random-effect model was used.

**Figure 4:**
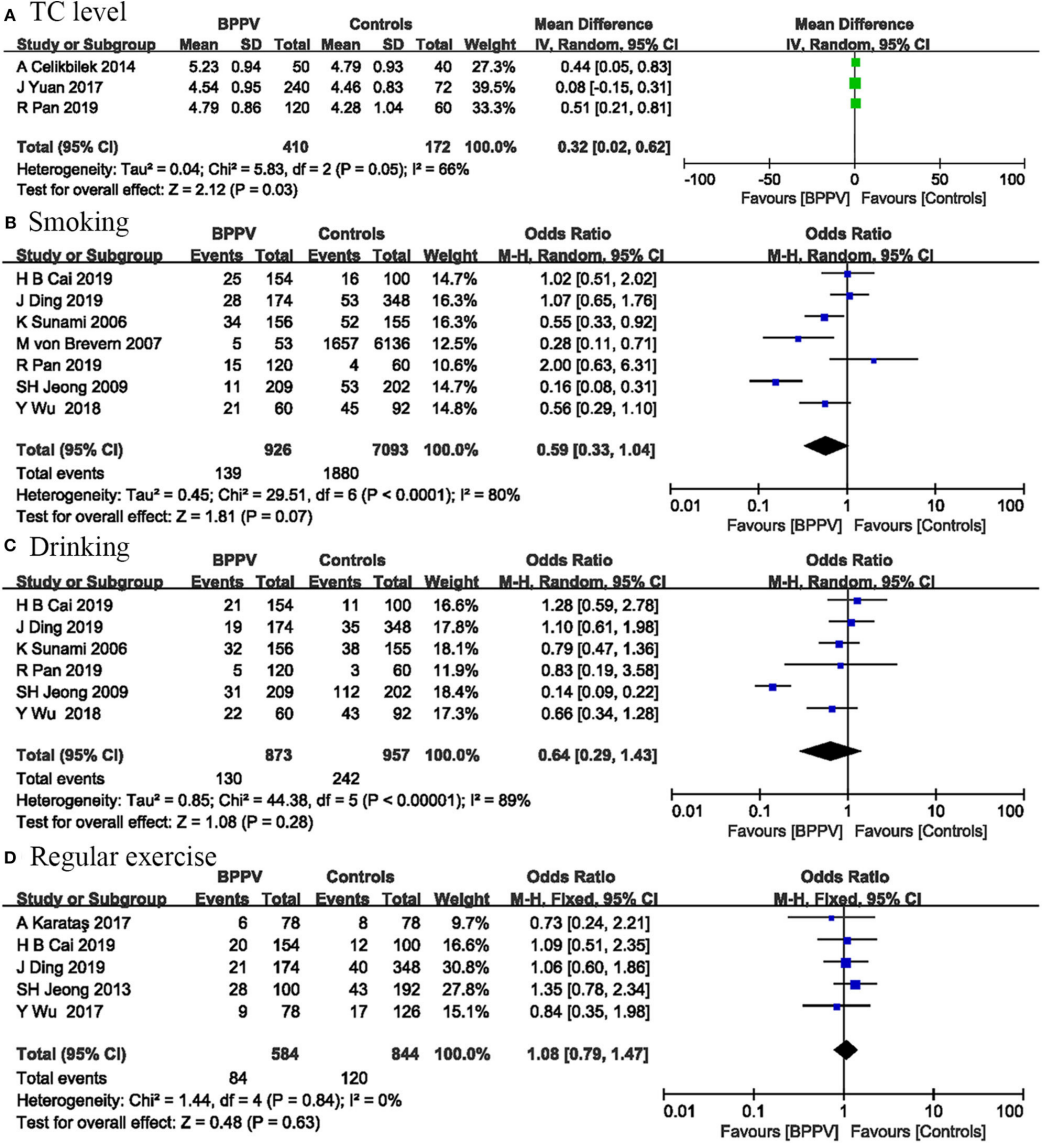
Forest plot of TC level **(A)** and changeable lifestyles including smoking **(B)**, drinking **(C)**, and regular exercise **(D)**.

Three studies involving 582 participants measured total cholesterol level to assess their influence on BPPV occurrence. The pooled evidence showed that patients with BPPV have a higher TC level than controls (MD = 0.32; 95% CI, 0.02–0.62; *P* = 0.03) (Figure 4). The *I*^2^-value was 66%, indicating significant heterogeneity between these studies.

### Changeable Lifestyles

Seven studies including 8,019 participants were conducted on the relationship between smoking and BPPV. The pooled results indicated that smoking was not associated with BPPV occurrence (OR = 0.59; 95% CI, 0.33–1.04; *P* = 0.07) (Figure 4). Statistical heterogeneity was significant (*I*^2^ = 80%; *P* < 0.0001).

Correlations between drinking and BPPV occurrence were performed in six studies involving 1,830 participants. No significant association was found between drinking and BPPV (OR = 0.64; 95% CI, 0.29–1.43; *P* = 0.28) (Figure 4). The *I*^2^-value was 89%, suggesting significant heterogeneity among included studies.

Five studies including 1,428 participants evaluated the effects of regular exercise on BPPV. Our analysis suggested that physical inactivity was not associated with BPPV occurrence (OR = 1.08; 95% CI, 0.79–1.47; *P* = 0.63) (Figure 4). There was no heterogeneity among these studies (*I*^2^ = 0%; *P* = 0.84).

## Discussion

This systematic review and meta-analysis indicated that female gender, vitamin D deficiency, osteoporosis, migraine, head trauma, and high TC level were risk factors for the occurrence of BPPV. There was no sufficient evidence to suggest that age, osteopenia, stroke, HTN, DM, hyperlipidemia, smoking, drinking, and physical inactivity were associated with BPPV occurrence. The accuracy of some of our results may be limited to significant heterogeneity or the limited number of included studies, so further research was needed to confirm some of our results.

Although many included studies controlled the sex ratio between the experimental and control groups, our analysis showed that women were more likely to develop BPPV than men. Previous studies have also suggested that women had a higher incidence of BPPV than in men, especially in the elderly women (4). This relationship may be related to estrogen deficiency in postmenopausal women, as estrogen may promote the development of osteoporosis and even BPPV (18). In addition, women BPPV patients have a higher risk of recurrence than men (9, 31). Therefore, further research between estrogen levels and BPPV may help early diagnosis and prevention of BPPV.

Our analysis of serum vitamin D level suggested that vitamin D deficiency appeared to be a risk factor for the occurrence of BPPV. This result was consistent with a previous meta-analysis (32). BPPV significantly increased the risk of fractures and osteoporosis, which may be related to vitamin D deficiency in BPPV patients (33, 34). Moreover, serum vitamin D level can be affected by estrogen deficiency (35), which may help explain why BPPV was more common in postmenopausal women. Thus, serum vitamin D level may be used for the auxiliary diagnosis of atypical BPPV as a serum predictor. In addition, some studies showed that vitamin D supplements can effectively improve symptoms of patients with BPPV (36) and have preventive effects on BPPV recurrence (37). Hence, vitamin D supplements may have important effects on improving the diagnosis and prognosis of patients with BPPV.

Our analysis results indicated that osteoporosis was a risk factor for BPPV occurrence, but osteopenia was not. A previous systematic review also showed that BPPV may be associated with osteoporosis or osteopenia (38). Many studies suggested that bone mineral density values in BPPV patients were lower than those in controls (39). In addition, osteoporosis and osteopenia may also be associated with BPPV recurrence (40, 41). Thus, treatment of osteoporosis may help prevent the occurrence of BPPV and improve the prognosis of BPPV patients (42). Further studies were needed to determine the effects of BMD on BPPV occurrence and recurrence.

The pooled results showed that BPPV has no significant relationship with hyperlipidemia, but BPPV patients have a higher TC level. An increased TC level was a risk factor for BPPV occurrence. A higher TC level or hyperlipidemia can cause vascular damage in the inner ear, which may lead to BPPV occurrence (4). In addition, a recent study found that the three rs2074880 genotypes in the CACNA1A (Calcium Voltage-Gated Channel Subunit Alpha1 A) gene were associated with increased levels of cholesterol in BPPV patients (28). The relationship between TC level and BPPV has not been adequately studied. Further studies were required to confirm these results.

BPPV was frequently induced by secondary factors such as head trauma, migraine, or other inner ear diseases. Recent studies showed that migraine (43) and head trauma (29) were significantly associated with an increased incidence of BPPV. Most included studies excluded patients with any history of vestibular or neurological diseases, including head trauma and migraine. Our analysis still showed that migraine and head trauma were risk factors for BPPV occurrence. However, the limited number of studies included or significant heterogeneity may limit the accuracy of these results.

Some studies have investigated associations between vascular risk factors and BPPV, such as hypertension, DM, and hyperlipidemia, but the results were controversial (4, 19). In addition, vascular comorbidities may also be risk factors for BPPV recurrence (8, 9). However, our analysis showed that migraine and high TC level were risk factors for BPPV occurrence, while HTN, DM, hyperlipidemia, and stroke were not. The limited number of eligible studies or significant heterogeneity among studies may limit the accuracy of these results. Large-scale studies of these risk factors were needed to confirm the reliability of these results.

Previous studies suggested that smoking has adverse effects on middle ear diseases and hearing loss (44) and even makes the treatment of vertigo ineffective (45). However, some studies have shown that smoking can reduce the incidence of BPPV, prevent the recurrence of BPPV, and shorten the recovery time of BPPV (16). The relationship between smoking and BPPV was quite controversial and had not been adequately investigated. We expected that smoking was a potential risk factor for BPPV occurrence, but pooled results showed no significant relationship between smoking and BPPV occurrence. Significant heterogeneity among studies may limit the accuracy of this results. Further investigations were needed to establish the effects of smoking on BPPV.

Our analysis showed no significant association between BPPV and physical inactivity. However, previous studies showed that moderate physical exercise can prevent the occurrence of BPPV and decrease the risk of falls and fractures, especially in the elderly (46). Intense physical activity may trigger posttraumatic BPPV without head trauma (47), but a study showed that BPPV caused by intense physical activity was a rare condition (48). Some included studies did not give specific definition, which may limit the accuracy of this result. The role of regular exercise and moderate exercise in BPPV needed further investigations.

## Limitations

Inevitably, there were several limitations in this meta-analysis. First, searches were restricted to English literature, which means that potentially high-quality literature may not be included in our analysis. Second, some potential risk factors were not analyzed in our analysis, because too few published studies were available, such as coronary heart disease, serum uric acid level, and albumin level. Third, subgroup analysis of each risk factor was not performed due to insufficient data. Furthermore, many included studies were retrospectively conducted in Asia and BPPV had many levels of its severity, which may limit the reliability of our results. In addition, for some risk factors, the limited number of included studies, significant heterogeneity, or ambiguous definition may limit the accuracy of these results. Large-scale randomized controlled trial (RCT) studies were necessary to confirm the reliability of our results.

## Conclusion

This meta-analysis was based on 19 studies involving a total of 14,286 participants, which provided strong evidence that female gender, vitamin D deficiency, osteoporosis, high TC level, migraine, and head trauma were risk factors for the occurrence of BPPV. However, the effects of other risk factors on BPPV occurrence needed further investigations. Further investigations should focus on exploring potential mechanisms, how to effectively intervene in high-risk populations, and preventing these risk factors as much as possible.

## Data Availability Statement

All datasets presented in this study are included in the article/Supplementary Material.

## Author Contributions

JC and WZ contributed to literature search, data analysis, and drafting and revision of the manuscript. JC and XY contributed to data collection and crafting and revision of the tables and figures. PZ given constructive suggestions for the revision of this manuscript. All authors contributed to the article and approved the submitted version.

## Conflict of Interest

The authors declare that the research was conducted in the absence of any commercial or financial relationships that could be construed as a potential conflict of interest.

## References

[1] Hanley K O'Dowd T Considine N. A systematic review of vertigo in primary care. Br J Gen Pract. (2001) 51:666–71. 11510399 PMC1314080

[2] Neuhauser HK von Brevern M Radtke A Lezius F Feldmann M Ziese T. Epidemiology of vestibular vertigo: a neurotologic survey of the general population. Neurology. (2005) 65:898–904. 10.1212/01.wnl.0000175987.59991.3d 16186531

[3] Kim JS Zee DS. Clinical practice. Benign paroxysmal positional vertigo. N Engl J Med. (2014) 370:1138–47. 10.1056/NEJMcp1309481 24645946

[4] von Brevern M Radtke A Lezius F Feldmann M Ziese T Lempert T. Epidemiology of benign paroxysmal positional vertigo: a population based study. J Neurol Neurosurg Psychiatry. (2007) 78:710–5. 10.1136/jnnp.2006.100420 17135456 PMC2117684

[5] Liao WL Chang TP Chen HJ Kao CH. Benign paroxysmal positional vertigo is associated with an increased risk of fracture: a population-based cohort study. J Orthop Sports Phys Ther. (2015) 45:406–12. 10.2519/jospt.2015.5707 25808526

[6] Kao CL Cheng YY Leu HB Chen TJ Ma HI Chen JW. Increased risk of ischemic stroke in patients with benign paroxysmal positional vertigo: a 9-year follow-up nationwide population study in Taiwan. Front Aging Neurosci. (2014) 6:108. 10.3389/fnagi.2014.00108 24917815 PMC4040439

[7] Lo MH Lin CL Chuang E Chuang TY Kao CH. Association of dementia in patients with benign paroxysmal positional vertigo. Acta Neurol Scand. (2017) 135:197–203. 10.1111/ane.12581 26932875

[8] De Stefano A Dispenza F Suarez H Perez-Fernandez N Manrique-Huarte R Ban JH. A multicenter observational study on the role of comorbidities in the recurrent episodes of benign paroxysmal positional vertigo. Auris Nasus Larynx. (2014) 41:31–6. 10.1016/j.anl.2013.07.007 23932347

[9] Zhu CT Zhao XQ Ju Y Wang Y Chen MM Cui Y. Clinical characteristics and risk factors for the recurrence of benign paroxysmal positional vertigo. Front Neurol. (2019) 10:1190. 10.3389/fneur.2019.01190 31798518 PMC6863975

[10] Işik GÇ Cevik Y Emektar E Corbacioglu S Analysis of vitamin D and calcium levels in benign paroxysmal positional vertigo. Eurasian J Emerg Med. (2017) 16:128–32. 10.5152/eajem.2017.58077

[11] Wu Y Fan Z Jin H Guan Q Zhou M Lu X. Assessment of bone metabolism in male patients with benign paroxysmal positional vertigo. Front Neurol. (2018) 9:742. 10.3389/fneur.2018.00742 30233488 PMC6135048

[12] Karataş A Acar Yüceant G Yüce T Haci C Cebi IT Salviz M. Association of benign paroxysmal positional vertigo with osteoporosis and vitamin D deficiency: a case controlled study. J Int Adv Otol. (2017) 13:259–65. 10.5152/iao.2016.2640 28274898

[13] Chang TP Lin YW Sung PY Chuang HY Chung HY Liao WL. Benign paroxysmal positional vertigo after dental procedures: a population-based case-control study. PLoS ONE. (2016) 11:e0153092. 10.1371/journal.pone.0153092 27044009 PMC4820237

[14] Yang CJ Kim Y Lee HS Park HJ. (2018). Bone mineral density and serum 25-hydroxyvitamin D in patients with idiopathic benign paroxysmal positional vertigo. J Vestib Res. (2018) 27:287–94. 10.3233/VES-170625 29400685

[15] Jeong SH Kim JS Shin JW Kim S Lee H Lee AY. Decreased serum vitamin D in idiopathic benign paroxysmal positional vertigo. J. Neurol. (2013) 260:832–8. 10.1007/s00415-012-6712-2 23096068

[16] Sunami K Tochino R Tokuhara Y Yamamoto H Tomita S Koshimo N. Effects of cigarettes and alcohol consumption in benign paroxysmal positioning vertigo. Acta Otolaryngol. (2006) 126:834–8. 10.1080/00016480500527474 16846926

[17] Cai HB Duan L Tian T Li ZC Zhao CC Ge ZM. Elevated serum macrophage migration inhibitory factor levels correlate with benign paroxysmal positional vertigo and recurrence events. Biosci. Rep. (2019) 39:BSR20191831 10.1042/BSR20191831 31406010 PMC6706593

[18] Yang H Gu H Sun W Li Y Wu H Burnee M. Estradiol deficiency is a risk factor for idiopathic benign paroxysmal positional vertigo in postmenopausal female patients. Laryngoscope. (2018) 128:948–53. 10.1002/lary.26628 28480516

[19] Zhang D Zhang S Zhang H Xu Y Fu S Yu M. Evaluation of vertebrobasilar artery changes in patients with benign paroxysmal positional vertigo. NeuroReport. (2013) 24:741–5. 10.1097/WNR.0b013e328364b948 23903461

[20] Yuan J Dai J Li WA Hu W. (2017). Factors associated with benign paroxysmal positional vertigo: a chinese case-control study. Med Sci Monit. (2017) 23:3885–9. 10.12659/MSM.905716 28800356 PMC5565235

[21] Ziavra NV Bronstein AM. Is uric acid implicated in benign paroxysmal positional vertigo? J Neurol. (2004) 251:115. 10.1007/s00415-004-0277-7 14999502

[22] Han W Fan Z Zhou M Guo X Yan W Lu XZ. Low 25-hydroxyvitamin D levels in postmenopausal female patients with benign paroxysmal positional vertigo. Acta Otolaryngol. (2018) 138:443–6. 10.1080/00016489.2017.1416168 29272984

[23] Jeong SH Choi SH Kim JY Koo JW Kim HJ Kim JS. Osteopenia and osteoporosis in idiopathic benign positional vertigo. Neurology. (2009) 72:1069–76. 10.1212/01.wnl.0000345016.33983.e0 19307540

[24] Wu Y Gu C Han W Lu X Chen C Fan Z. (2017). Reduction of bone mineral density in native Chinese female idiopathic benign paroxysmal positional vertigo patients. Am J Otolaryngol. (2017) 39:31–3. 10.1016/j.amjoto.2017.09.004 29042068

[25] Ding J Liu L Kong WK Chen XB Liu X. Serum levels of 25-hydroxy vitamin D correlate with idiopathic benign paroxysmal positional vertigo. Biosci Rep. (2019) 39:BSR20190142. 10.1042/BSR20190142 30962270 PMC6488856

[26] Celikbilek A Gencer ZK Saydam L Zararsiz G Tanik N Ozkiris M. Serum uric acid levels correlate with benign paroxysmal positional vertigo. Eur J Neurol. (2014) 21:79–85. 10.1111/ene.12248 23952220

[27] Stang A. (2010). Critical evaluation of the Newcastle-Ottawa scale for the assessment of the quality of nonrandomized studies in meta-analyses. Euro J Epidemiol. (2010) 25:603–5. 10.1007/s10654-010-9491-z 20652370

[28] Pan R Qi X Wang F Chong Y Li X Chen Q. Correlations of calcium voltage-gated channel subunit alpha1 A (CACNA1A) Gene Polymorphisms with Benign Paroxysmal Positional Vertigo. Med Sci Monit. (2019) 25:946–51. 10.12659/MSM.912359 30710491 PMC6368824

[29] Kim M Lee DS Hong TH, Joo Cho H. Risk factor of benign paroxysmal positional vertigo in trauma patients: a retrospective analysis using Korean trauma database. Medicine. (2018) 97:e13150 10.1097/MD.0000000000013150 30544375 PMC6310538

[30] Higgins JP Thompson SG Deeks JJ Altman DG. Measuring inconsistency in meta-analyses. BMJ. (2003) 327:557–60. 10.1136/bmj.327.7414.557 12958120 PMC192859

[31] Luryi AL Lawrence J Bojrab DI LaRouere M Babu S Zappia J Recurrence in benign paroxysmal positional vertigo: a large, single-institution study. Otol Neurotol. (2018) 39:622–7. 10.1097/MAO.0000000000001800 29649052

[32] Yang B Lu Y Xing D Zhong W Tang Q Liu J. Association between serum vitamin D levels and benign paroxysmal positional vertigo: a systematic review and meta-analysis of observational studies. Eur Arch Otorhinolaryngol. (2020) 277:169–77. 10.1007/s00405-019-05694-0 31630244

[33] Lawson J Bamiou DE Cohen HS Newton J. Positional vertigo in a falls service. Age Ageing. (2008) 37:585–9. 10.1093/ageing/afn151 18664517

[34] Deandrea S Lucenteforte E Bravi F Foschi R La Vecchia C Negri. Risk factors for falls in community-dwelling older people: a systematic review and meta-analysis. Epidemiology. (2010) 21:658–68. 10.1097/EDE.0b013e3181e89905 20585256

[35] Yu S Fang H Han J Cheng X Xia L Li S The high prevalence of hypovitaminosis D in China: a multicenter vitamin D status survey. Medicine. (2015) 94:e585 10.1097/MD.0000000000000585 25715263 PMC4554140

[36] Gu X Dong F Gu J. (2018). Analysis of effect of 1α-hydroxyvitamin D3 on benign paroxysmal positional vertigo and risk factors. Exp Ther Med. (2018) 15:2321–6. 10.3892/etm.2018.5699 29456639 PMC5795805

[37] Buki B Ecker M Junger H Lundberg YW. Vitamin D deficiency and benign paroxysmal positioning vertigo. Med Hypotheses. (2013) 80:201–4. 10.1016/j.mehy.2012.11.029 23245911 PMC4196321

[38] Yu S Liu F Cheng Z Wang Q. Association between osteoporosis and benign paroxysmal positional vertigo: a systematic review. BMC Neurol. (2014) 14:110. 10.1186/1471-2377-14-110 24886504 PMC4039044

[39] Jang YS Kang MK. Relationship between bone mineral density and clinical features in women with idiopathic benign paroxysmal positional vertigo. Otol Neurotol. (2009) 30:95–100. 10.1097/MAO.0b013e31818f5777 19008769

[40] Kim SY Han SH Kim YH Park MH. Clinical features of recurrence and osteoporotic changes in benign paroxysmal positional vertigo. Auris Nasus Larynx. (2017) 44:156–61. 10.1016/j.anl.2016.06.006 27423924

[41] Yamanaka T Shirota S Sawai Y Murai T Fujita N Hosoi H. Osteoporosis as a risk factor for the recurrence of benign paroxysmal positional vertigo. Laryngoscope. (2013) 123:2813–6. 10.1002/lary.24099 23568754

[42] Mikulec AA Kowalczyk KA Pfitzinger ME Harris DA Jackson LE. Negative association between treated osteoporosis and benign paroxysmal positional vertigo in women. J Laryngol Otol. (2010) 124:374–6. 10.1017/S002221510999209X 19930786

[43] Kim SK Hong SM Park IS Choi HG. Association between migraine and benign paroxysmal positional vertigo among adults in South Korea. JAMA Otolaryngol Head Neck Surg. (2019) 145:307–12. 10.1001/jamaoto.2018.4016 30676633 PMC6481427

[44] Gaur K Kasliwal N Gupta R. Association of smoking or tobacco use with ear diseases among men: a retrospective study. Tob Induc Dis. (2012) 10:4. 10.1186/1617-9625-10-4 22471960 PMC3366886

[45] Lin CY Young YH. Effect of smoking on the treatment of vertigo. Otol Neurotol. (2001) 22:369–72. 10.1097/00129492-200105000-00016 11347641

[46] Bazoni JA Mendes WS Meneses-Barriviera CL Melo JJ Costa Vde S Teixeira Dde C (2014). Physical activity in the prevention of benign paroxysmal positional vertigo: probable association. Int Arch Otorhinolaryngol. (2014) 18:387–90. 10.1055/s-0034-1384815 25992128 PMC4296996

[47] Vibert D Redfield RC Hausler R. Benign paroxysmal positional vertigo in mountain bikers. Ann Otol Rhinol Laryngol. (2007) 116:887–90. 10.1177/000348940711601203 18217506

[48] Giacomini PG Ferraro S Di Girolamo S Villanova I Ottaviani F. Benign paroxysmal positional vertigo after intense physical activity: a report of nine cases. Eur Arch Otorhinolaryngol. (2009) 266:1831–5. 10.1007/s00405-009-0938-3 19288124

