# Supplementary material for: Risk Factors for the Occurrence of Benign Paroxysmal Positional Vertigo: A Systematic Review and Meta-Analysis
Source: Front Neurol. 2020 Jun 23;11:506. doi: 10.3389/fneur.2020.00506 (PMC7324663; doi:10.3389/fneur.2020.00506)
Supplement: Supplementary file 1 [file Data_Sheet_1.docx]

**Risk factors for the occurrence of Benign Paroxysmal Positional Vertigo: A Systematic Review and Meta-analysis**

***ONLINE SUPPLEMENT***

**Supplemental Figure 1:** A flow diagram showing the literature selection process.

**Supplemental Figure 2-5:** Funnel plots of some included risk factors.

**Supplemental Figure 6:** Sensitivity analysis of serum vitamin D level.

**Supplemental Table 1:** Quality assessment of each Study Included in this Meta-Analysis.

**Supplemental Figure 1**


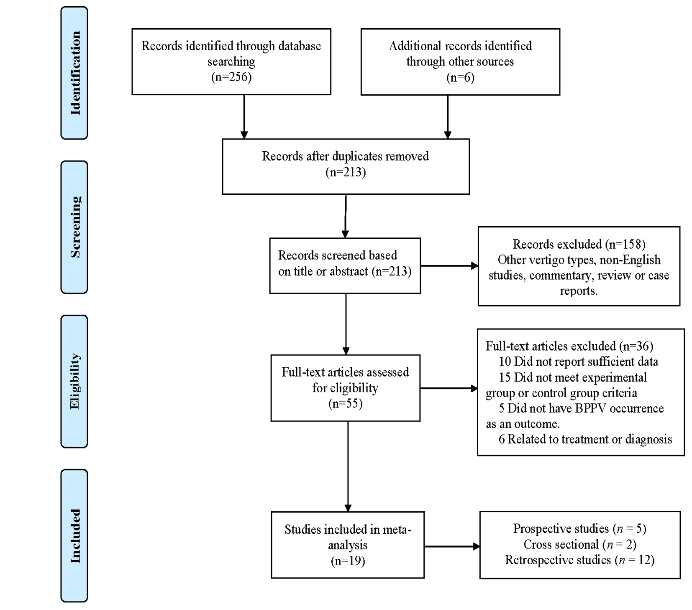


**Supplemental Figure 2-5** (F2-5: Female gender, Age, Hypertension, Diabetes mellitus)


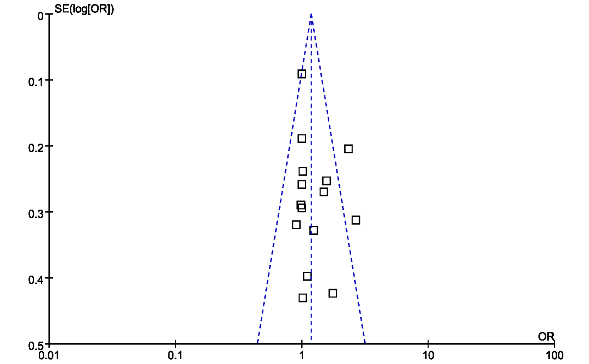

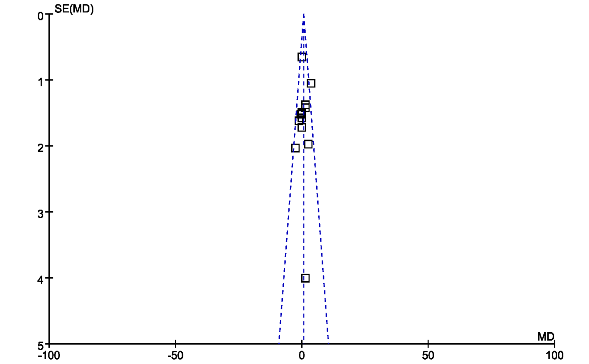


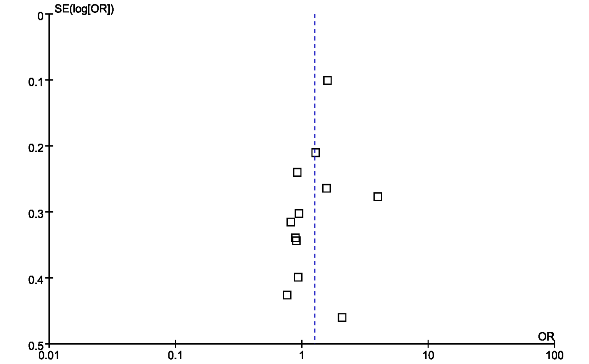

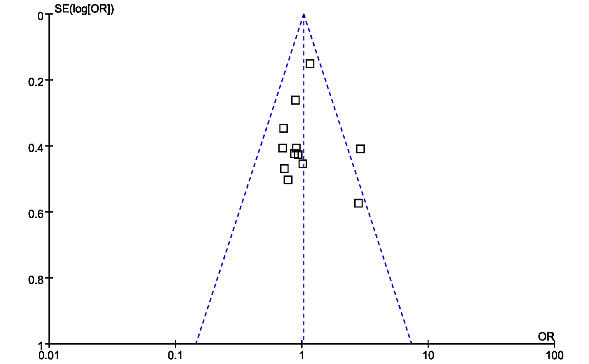


**Supplemental Figure 5**


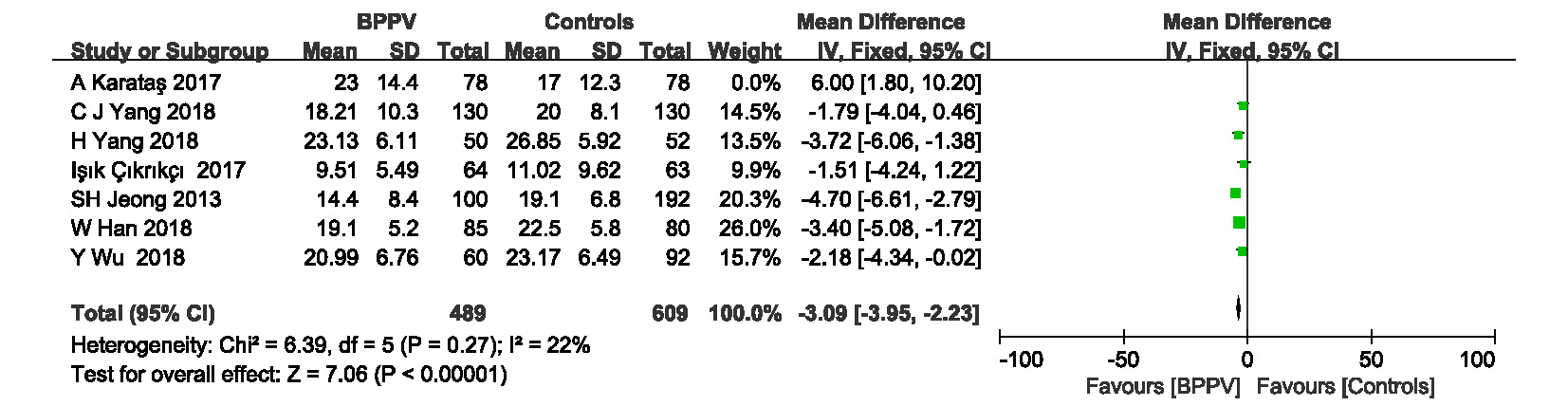


**Supplemental Table 1: Quality assisment of each Study Included in this Meta-Analysis**

| Study | Selection | | | | Comparability | | Exposure | | | **Score** |
| --- | --- | --- | --- | --- | --- | --- | --- | --- | --- | --- |
|  | Validation of the case status | Representativeness of the case group | Selection of the control group | Definition of the control group | Matching the most important factor | Adjustment of confounding factors | Assessment of exposure | Consistency of exposure assessment methods | comparable non-response rate | Total scores |
| A Karataş | 1 | 0 | 0 | 1 | 1 | 1 | 1 | 1 | 1 | 7 |
| J Yuan | 1 | 1 | 0 | 0 | 1 | 1 | 1 | 1 | 1 | 7 |
| A Celikbilek | 1 | 0 | 0 | 0 | 1 | 1 | 1 | 1 | 1 | 6 |
| C J Yang | 0 | 1 | 0 | 1 | 1 | 1 | 1 | 1 | 1 | 7 |
| Işık Çıkrıkçı | 1 | 0 | 0 | 1 | 1 | 0 | 1 | 1 | 1 | 6 |
| H B Cai | 1 | 0 | 0 | 1 | 1 | 0 | 0 | 1 | 1 | 5 |
| SH Jeong | 1 | 1 | 1 | 1 | 1 | 1 | 1 | 1 | 1 | 9 |
| J Ding | 1 | 0 | 0 | 1 | 1 | 1 | 1 | 1 | 1 | 7 |
| M von Brevern | 1 | 1 | 1 | 1 | 0 | 0 | 0 | 0 | 1 | 5 |
| SH Jeong | 1 | 1 | 0 | 1 | 1 | 1 | 1 | 1 | 1 | 8 |
| W Han | 1 | 0 | 0 | 1 | 0 | 1 | 1 | 1 | 1 | 6 |
| Y Wu | 1 | 0 | 0 | 1 | 0 | 1 | 1 | 1 | 1 | 6 |
| Y Wu | 1 | 0 | 0 | 1 | 1 | 1 | 1 | 1 | 1 | 7 |
| D Zhang | 1 | 0 | 0 | 1 | 1 | 1 | 0 | 1 | 0 | 5 |
| H Yang | 1 | 0 | 0 | 1 | 1 | 1 | 1 | 1 | 1 | 7 |
| T P Chang | 1 | 1 | 1 | 1 | 1 | 1 | 1 | 1 | 1 | 9 |
| K Sunami | 1 | 0 | 0 | 1 | 1 | 0 | 1 | 1 | 1 | 6 |
| R Pan | 1 | 1 | 0 | 1 | 1 | 1 | 1 | 1 | 1 | 8 |
| Kim M | 1 | 1 | 1 | 1 | 0 | 1 | 1 | 1 | 0 | 7 |
